# Diversity of Sea Star-Associated Densoviruses and Transcribed Endogenous Viral Elements of Densovirus Origin

**DOI:** 10.1128/JVI.01594-20

**Published:** 2020-12-09

**Authors:** Elliot W. Jackson, Roland C. Wilhelm, Mitchell R. Johnson, Holly L. Lutz, Isabelle Danforth, Joseph K. Gaydos, Michael W. Hart, Ian Hewson

**Affiliations:** a Department of Microbiology, Cornell University, Ithaca, New York, USA; b School of Integrative Plant Sciences, Bradfield Hall, Cornell University, Ithaca, New York, USA; c Department of Pediatrics, School of Medicine, University of California San Diego, La Jolla, California, USA; d Scripps Institution of Oceanography, University of California San Diego, La Jolla, California, USA; e SeaDoc Society, UC Davis Karen C. Drayer Wildlife Health Center—Orcas Island Office, Eastsound, Washington, USA; f Department of Biological Sciences, Simon Fraser University, Burnaby, British Columbia, Canada; University of Texas Southwestern Medical Center

**Keywords:** densovirus, parvovirus, sea star wasting disease, viral discovery, viral metagenomics, ssDNA viruses

## Abstract

A viral etiology of sea star wasting syndrome (SSWS) was originally explored with virus-sized material challenge experiments, field surveys, and metagenomics, leading to the conclusion that a densovirus is the predominant DNA virus associated with this syndrome and, thus, the most promising viral candidate pathogen. Single-stranded DNA viruses are, however, highly diverse and pervasive among eukaryotic organisms, which we hypothesize may confound the association between densoviruses and SSWS. To test this hypothesis and assess the association of densoviruses with SSWS, we compiled past metagenomic data with new metagenomic-derived viral genomes from sea stars collected from Antarctica, California, Washington, and Alaska. We used 179 publicly available sea star transcriptomes to complement our approaches for densovirus discovery. Lastly, we focus the study on sea star-associated densovirus (SSaDV), the first sea star densovirus discovered, by documenting its biogeography and putative tissue tropism. Transcriptomes contained only endogenized densovirus elements similar to the NS1 gene, while numerous extant densoviral genomes were recovered from viral metagenomes. SSaDV was associated with nearly all tested species from southern California to Alaska, and in contrast to previous work, we show that SSaDV is one genotype among a high diversity of densoviruses present in sea stars across the West Coast of the United States and globally that are commonly associated with grossly normal (i.e., healthy or asymptomatic) animals. The diversity and ubiquity of these viruses in sea stars confound the original hypothesis that one densovirus is the etiological agent of SSWS.

**IMPORTANCE** The primary interest in sea star densoviruses, specifically SSaDV, has been their association with sea star wasting syndrome (SSWS), a disease that has decimated sea star populations across the West Coast of the United States since 2013. The association of SSaDV with SSWS was originally drawn from metagenomic analysis, which was further studied through field surveys using quantitative PCR (qPCR), with the conclusion that it was the most likely viral candidate in the metagenomic data based on its representation in symptomatic sea stars compared to asymptomatic sea stars. We reexamined the original metagenomic data with additional genomic data sets and found that SSaDV was 1 of 10 densoviruses present in the original data set and was no more represented in symptomatic sea stars than in asymptomatic sea stars. Instead, SSaDV appears to be a widespread, generalist virus that exists among a large diversity of densoviruses present in sea star populations.

## Abstract (precis)

The primary interest in sea star densoviruses, specifically SSaDV, has been their association with sea star wasting syndrome (SSWS), a disease that has decimated sea star populations across the West Coast of the United States since 2013. The association of SSaDV with SSWS was originally drawn from metagenomic analysis, which was further studied through field surveys using quantitative PCR (qPCR), with the conclusion that it was the most likely viral candidate in the metagenomic data based on its representation in symptomatic sea stars compared to asymptomatic sea stars. We reexamined the original metagenomic data with additional genomic data sets and found that SSaDV was 1 of 10 densoviruses present in the original data set and was no more represented in symptomatic sea stars than in asymptomatic sea stars. Instead, SSaDV appears to be a widespread, generalist virus that exists among a large diversity of densoviruses present in sea star populations.

## INTRODUCTION

Single-stranded DNA (ssDNA) viruses are among the most diverse and prevalent group of viruses infecting eukaryotes, bacteria, and archaea (1–4). Recognition of their ubiquity has been made possible through the use of rolling-circle amplification, which preferentially amplifies circular nucleic acid templates prior to high-throughput sequencing (5, 6). As a result, ssDNA viruses that possess circular genomes are significantly overrepresented compared to those with linear genomes. There are currently nine established families of ssDNA viruses that infect eukaryotes, only two of which possess linear genomes—*Bidnaviridae* and *Parvoviridae* (7). While our knowledge of the circular ssDNA viruses has expanded tremendously, the discovery of linear ssDNA viruses has lagged. Collectively, the known viral diversity of these two families likely represents only a small proportion of actual extant diversity, particularly within the subfamily *Densovirinae* (family *Parvoviridae*).

Currently, 17 viral species are recognized by the International Committee on the Taxonomy of Viruses to belong to the subfamily *Densovirinae* (commonly referred to as densoviruses) (8). Densoviruses infect invertebrates and until recently were only known to infect insects (orders Blattodea, Diptera, Hemiptera, Hymenoptera, Lepidoptera, and Orthoptera) and decapod crustaceans (shrimp and crayfish) (9). Unlike for circular ssDNA virus groups, the discovery of novel linear densovirus genomes has occurred primarily via the use of classical methods, such as viral purification through cell culture or viral enrichment via an animal model followed by nucleic acid sequencing, but these classical methods are more recently supplemented by the use of high-throughput sequencing to explore viral diversity. Transcriptomic and metagenomic analyses have generated a growing body of evidence to suggest that densoviruses infect a more phylogenetically diverse array of invertebrate hosts outside the phylum Arthropoda (10–13). Insights into the evolution and biology of densoviruses may be found by expanding the host range of these viruses.

The 2014 discovery of densoviruses associated with sea stars and sea urchins (phylum Echinodermata) was a significant step toward expanding the range of densoviruses beyond arthropods (11, 14). The primary interest in echinoderm densoviruses has been their association with sea star wasting syndrome (SSWS; also referred to as sea star wasting disease and asteroid idiopathic wasting syndrome), which is an epidemic affecting sea stars on the East and West coasts of North America (11, 15). The gross signs of SSWS include lethargy and limb curling, loss in turgor, epidermal ulceration and tissue loss, ray autotomy, and eversion of viscera through the body wall, all of which generally lead to the animal’s death (11, 16). There is currently no clinical pathological case definition for SSWS, so a symptomatic individual is defined by the presentation of these gross signs (16). The etiology of SSWS is also currently unknown. A densovirus, commonly known as sea star-associated densovirus (SSaDV), was hypothesized to cause SSWS, but it has yet to be determined if SSaDV is a pathogen (11, 17). Originally, SSaDV was thought to be the etiological agent of SSWS observed in both Pacific and Atlantic sea star populations (11, 15). However, the recent discovery of a genetically similar densovirus associated with sea stars on the Atlantic coast, Asterias forbesi-associated densovirus (AfaDV), raises new questions about densovirus diversity and the putative role of densoviruses in SSWS (13). The discovery of AfaDV prompted us to explore the diversity of densoviruses at a greater geographic scale and to define the biogeography of SSaDV and expand our understanding of the ecology, evolution, and diversity of these viruses.

In this study, we employed a multiomic approach to document the biodiversity of densovirus populations in sea stars by first reassessing the original metagenomic data set leading to the association of densoviruses and SSWS. We used publicly available sea star transcriptomes/genomes and sea star viral metagenomes for viral discovery and PCR to document the prevalence, putative tissue tropism, and biogeography of SSaDV. We report the discovery of >30 novel sea star densoviruses associated with sea stars from the Southern Ocean around Antarctica (11 genomes) and from the temperate eastern Pacific (24 genomes), as well as the observation of numerous endogenized viral elements (EVEs) from sea star transcriptomes and genomes. We found that SSaDV putatively has a wide tissue tropism and is associated with sea stars in the eastern Pacific across a broad latitudinal range from southern California to Alaska, corroborating previous findings by Hewson et al. (11). The identification of SSaDV as one among many densoviruses infecting sea stars on the West Coast of the United States suggests that densoviruses may comprise a normal component of the sea star microbiome, bringing into question the association of densoviruses with SSWS.

## RESULTS

### Reanalysis of metagenomes published in the work of Hewson et al.

**FIG 1:**
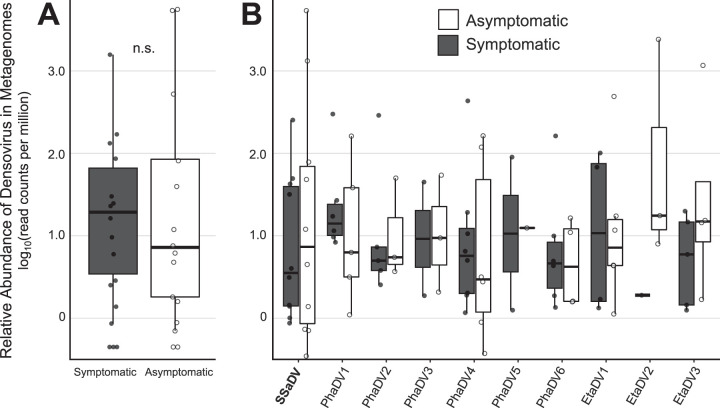
Reanalysis of metagenomic data presented by Hewson et al. (11). SSaDV is 1 of 10 densoviruses present in the data set and based on read mapping analysis (≥95% read identity) and is not more abundant in symptomatic than in asymptomatic individuals. (A) Relative abundance of all reads recruited to densovirus genomes. n.s., no significance, based on Welch two-sample *t* test (*P* = 0.7697, *df* = 25.137, *t* = −0.29592). (B) Read recruitment separated by densovirus genotype.

The reanalysis of the viral metagenomic data presented by Hewson et al. (11) led to the discovery of 9 additional densovirus genomes (Fig. 1 and Table 1). The densovirus contigs ranged in length from 3,391 to 6,053 nucleotides (nt) (5,002 ± 921 [mean ± standard deviation]). SSaDV was the only densovirus assembled into a complete or nearly complete genome across multiple metagenomes and had the highest read recruitment among all libraries (Table 1; see also Table S5 in the supplemental material). The previously published partial SSaDV genome (5,050 nt) lacked the NS3 open reading frame (ORF) and inverted terminal repeats (ITRs) (11). In this study, we recovered three SSaDV genomes of various sizes from 3 of the 32 metagenomes (Table 1). The largest of these genomes (6,053 nt) contained the expected ORFs (NS1, NS2, NS3, and VP), ITRs, and hairpins within the ITRs and therefore likely represents a complete genome. It is possible that the ITR region of the genome is not complete, due to challenges posed by assembling regions with high frequency of repeats using short-read technology. Members of the genus *Ambidensovirus* typically have ITRs of >500 nt, which is considerably longer than the ITR regions we observed (9). The ITRs in SSaDV were 260 nt long on both sides of the genome and contained canonical hairpin structures that were 223 nt and thermodynamically favorable (Δ*G* = −106.40).

**TABLE 1:** Sea star-associated densovirus genome characteristics and metadata

Host (sea star species)	Animal collection site(s)	State or province and country	Collection yr	Metaviriome	Virus abbreviation	Contig size (nt)	Avg fold coverage	Orientation	Ambidensovirus subgroup^*a*^	Viral species^*b*^	GenBank accession no.
*Pisaster ochraceus*	Santa Cruz	California, USA	2013	RNA	PoaDV1^*c*^	5,719	427	Ambisense	A	Asteroid ambidensovirus 2	MT733037
	Olympic National Park	Washington, USA	2013	RNA	PoaDV2^*c*^	5,840	63	Ambisense	A	Asteroid ambidensovirus 9	MT733038
	Santa Cruz	California, USA	2013	RNA	PoaDV3^*c*^	5,758	134	Ambisense	A	Asteroid ambidensovirus 10	MT733039
	Olympic National Park	Washington, USA	2013	RNA	PoaDV4^*c*^	5,469	19	Ambisense	B	Asteroid ambidensovirus 11	MT733040
	Olympic National Park	Washington, USA	2013	RNA	PoaDV5^*c*^	5,415	15	Ambisense	A	Asteroid ambidensovirus 12	MT733041
	Olympic National Park	Washington, USA	2013	RNA	PoaDV6^*c*^	5,340	27	Ambisense	A	Asteroid ambidensovirus 5	MT733042
	Olympic National Park	Washington, USA	2013	RNA	PoaDV7^*c*^	5,052	30	Ambisense	B	Asteroid ambidensovirus 13	MT733043
	Santa Cruz	California, USA	2013	RNA	PoaDV8^*c*^	5,584	64	Ambisense	A	Asteroid ambidensovirus 14	MT733044
	Santa Cruz	California, USA	2013	RNA	PoaDV9^*c*^	4,827	15	Ambisense	B	Asteroid ambidensovirus 7	MT733045
	Olympic National Park	Washington, USA	2013	RNA	PoaDV10^*c*^	3,264	18	Ambisense	NA	Asteroid ambidensovirus 15	MT733046
	Olympic National Park	Washington, USA	2013	RNA	PoaDV11^*c*^	5,095	51	Ambisense	B	Asteroid ambidensovirus 8	MT733047
	Santa Cruz	California, USA	2013	RNA	PoaDV12^*c*^	5,270	451	Ambisense	A	Asteroid ambidensovirus 6	MT733048
	Santa Cruz	California, USA	2018	RNA	PoaDV13	5,963	30	Ambisense	A	Asteroid ambidensovirus 16	MT733049
	Santa Cruz	California, USA	2018	RNA	PoaDV14	5,274	72	Ambisense	B	Asteroid ambidensovirus 17	MT733050
*Leptasteria sp.*	Palmer's Point and Pigeon Point	California, USA	2017	RNA	LhaDV1	3,956	35	Ambisense	NA	Asteroid ambidensovirus 7	MT733022
*Pycnopodia helianthoides*	Seattle Aquarium	Washington, USA	2013	RNA	PhaDV1^*d*^	5,665	47	Ambisense	A	Asteroid ambidensovirus 2	MT733031
	Seattle Aquarium	Washington, USA	2013	RNA	PhaDV2^*d*^	5,326	27	Ambisense	A	Asteroid ambidensovirus 5	MT733032
	Seattle Aquarium	Washington, USA	2013	RNA	PhaDV3^*d*^	4,168	15	Ambisense	NA	Asteroid ambidensovirus 18	MT733033
	Seattle Aquarium	Washington, USA	2013	RNA	SSaDV^*d*^	5,663	34	Ambisense	A	Asteroid ambidensovirus 1	
	Seattle Aquarium	Washington, USA	2013	RNA	PhaDV4^*d*^	5,485	58	Ambisense	A	Asteroid ambidensovirus 6	MT733034
	Seattle Aquarium	Washington, USA	2013	RNA	PhaDV5^*d*^	3,391	16	NA	A	Only VP gene	MT733035
	Seattle Aquarium	Washington, USA	2013	RNA	PhaDV6^*d*^	3,446	27	Ambisense	NA	Asteroid ambidensovirus 8	MT733036
	Burrard Inlet	British Columbia, Canada	2013	DNA	SSaDV^*d*^	6,053	246	Ambisense	A	Asteroid ambidensovirus 1	MT733051
*Evasterias troschelii*	Cape Roger Curtis	British Columbia, Canada	2013	DNA	SSaDV^*d*^	5,206	27	Ambisense	A	Asteroid ambidensovirus 1	
	Cape Roger Curtis	British Columbia, Canada	2013	DNA	EtaDV1^*d*^	5,601	15	Ambisense	B	Asteroid ambidensovirus 19	MT733014
	Cape Roger Curtis	British Columbia, Canada	2013	DNA	EtaDV2^*d*^	5,700	38	Ambisense	B	Asteroid ambidensovirus 20	MT733015
	Cape Roger Curtis	British Columbia, Canada	2013	DNA	EtaDV3^*d*^	5,460	22	Ambisense	B	Asteroid ambidensovirus 21	MT733016
*Neosmilaster georgianus*	Palmer Station	Antarctica	2017	RNA	NgaDV1	5,605	122	Ambisense	B	Asteroid ambidensovirus 3	MT733025
	Palmer Station	Antarctica	2017	RNA	NgaDV2	5,383	395	Ambisense	B	Asteroid ambidensovirus 3	MT733026
	Palmer Station	Antarctica	2017	RNA	NgaDV3	5,328	388	Ambisense	B	Asteroid ambidensovirus 22	MT733027
	Palmer Station	Antarctica	2017	RNA	NgaDV4	5,352	36,423	Ambisense	A	Asteroid ambidensovirus 23	MT733028
	Palmer Station	Antarctica	2017	RNA	NgaDV5	4,832	15	Ambisense	B	Asteroid ambidensovirus 4	MT733029
	Palmer Station	Antarctica	2017	RNA	NgaDV6	4,886	63	Ambisense	A	Asteroid ambidensovirus 24	MT733030
Labidiaster annulatus	Palmer Station	Antarctica	2017	RNA	LaaDV1	5,872	443	Ambisense	B	Asteroid ambidensovirus 25	MT733017
	Palmer Station	Antarctica	2017	RNA	LaaDV2	5,240	214	Ambisense	B	Asteroid ambidensovirus 4	MT733018
	Palmer Station	Antarctica	2017	RNA	LaaDV3	5,018	501	Ambisense	B	Asteroid ambidensovirus 26	MT733019
	Palmer Station	Antarctica	2017	RNA	LaaDV4	4,972	68	Ambisense	B	Asteroid ambidensovirus 27	MT733020
	Palmer Station	Antarctica	2017	RNA	LaaDV5	5,413	263	Ambisense	B	Asteroid ambidensovirus 3	MT733021
Luidia maculata	Hong Kong	China	2014	DNA	LmaDV1^*c*^	5,446	16	Ambisense	B	Asteroid ambidensovirus 28	MT733023
Astropecten polyacanthus	Hong Kong	China	2014	DNA	ApaDV1^*c*^	3,061	8	Ambisense	NA	Asteroid ambidensovirus 29	MT733013
Mediaster aequalis	Ketchikan	Alaska, USA	2016	RNA	MaaDV1	5,956	1,220	Ambisense	A	Asteroid ambidensovirus 2	MT733024
*Asterias forbesi*	Nahant	Massachusetts, USA	2015	DNA	AfaDV	6,089	454	Ambisense	A	Asteroid ambidensovirus 1	MN190158

a Ambidensovirus subgroups defined in reference 19.

b Viral species defined by a pairwise amino acid sequence identity of NS1 proteins of >85% (18).

c Viral genotypes discovered from viral metagenomes prepared as described in reference 16.

d Viral genotypes discovered from viral metagenomes prepared as described in reference 11.

### SSaDV biogeography and tissue tropism.

**FIG 2:**
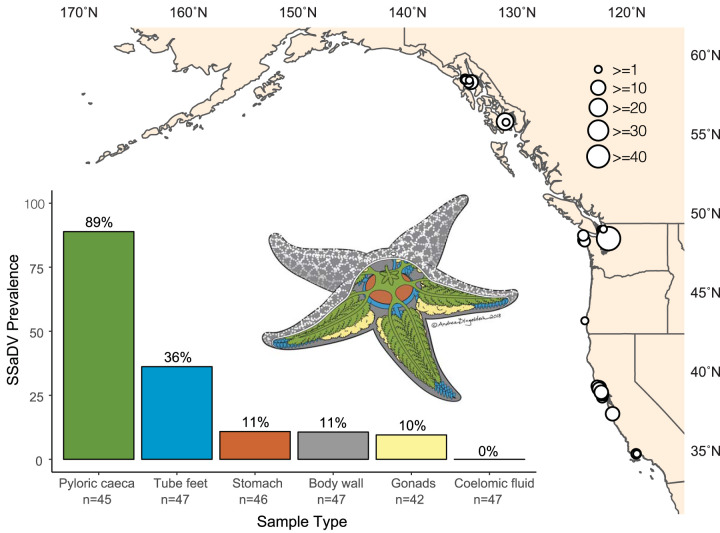
SSaDV is broadly distributed across the northeastern Pacific Ocean and putatively has a wide tissue tropism. White dots on map indicated PCR-positive samples, and the size of the dot corresponds to total number of PCR-positive samples at each site. Tissue tropism was assessed from 3 sea star species collected from one site (Langley Harbor, WA). The color of each bar corresponds to the anatomical region in the sea star illustration. Prevalence is defined as the number of PCR-positive samples divided by the total number of samples tested for each tissue.

A total of 148 of 660 animals were positive for SSaDV based on the PCR assay, equating to a global prevalence of 22.4% (Fig. 2 and Table S1). No samples were PCR positive from tissues collected in 2005. A total of 126 of 148 PCR amplicons were successfully Sanger sequenced, all confirming the specificity of the PCR assay (Table S1). Of the 148 SSaDV-positive sea stars, 22 were symptomatic (i.e., SSWS affected) and 126 were asymptomatic sea stars (Table S1). SSaDV was detected in 25 of the 42 locations that spanned a broad latitudinal range in the eastern Pacific from southern California to southeastern Alaska (Fig. 2 and Table S2). Nine of 12 sea star species tested positive, which include the following species (virus positive/sample total): Pisaster ochraceus (87/287), Pisaster brevispinus (10/10), Pisaster giganteus (3/9), Pycnopodia helianthoides (4/72), Evasterias troschelii (26/100), Dermasterias imbricata (8/34), a *Henricia* sp. (3/18), a *Leptasterias* sp. (6/42), and Patiria miniata (1/85). Only one individual was tested for each of the three species in which SSaDV was not detected. These included Orthasterias koehleri, Pteraster tesselatus, and Solaster stimpsoni.

Fine dissections of *Pisaster ochraceus* (*n* = 26 individuals), *Evasterias troschelii* (*n* = 11), and *Pisaster brevispinus* (*n* = 10) collected from Langley Harbor, WA, were used to assess putative tissue tropism. The viral prevalence among tissues was calculated by the number of tissues positive divided by the total number of tissues collected between these three species. SSaDV was detected most frequently in the pyloric caeca (89% [40/45]), followed by tube feet (36% [17/47]), stomach (11% [5/46]), body wall (11% [5/47]), and gonads (10% [4/42]) (Fig. 2 and Table S2). Similar to AfaDV, SSaDV was not detected in the coelomic fluid (0% [0/47]) (13).

### Genome discovery, genome comparison, motif annotation, and phylogeny.

**FIG 3:**
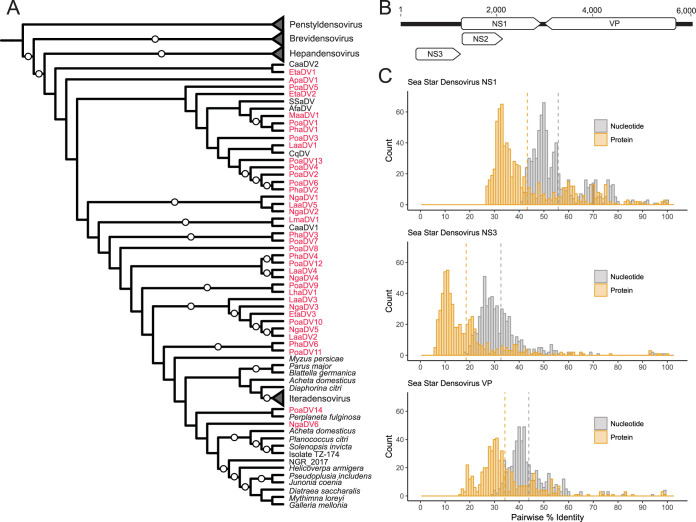
Sea star-associated densoviruses are genetically diverse and are not monophyletic. (A) Cladogram of a maximum likelihood phylogenetic tree of densoviruses based on alignment of amino acid sequences from the NS1 gene. Collapsed nodes represent densovirus genera, while all other branches belong to the genus *Ambidensovirus*. Red names indicate genomes discovered in this study. White circles represent 90 to 100% bootstrap support. (B) Representative densovirus genome showing genome organization. (C) Histograms of nucleotide and amino acid pairwise identity comparisons between all sea star-associated densoviruses for NS1, NS3, and VP ORFs. Dashed lines indicate mean pairwise identity.

An additional 29 densovirus genomes were recovered from newly prepared and reanalyzed sea star metagenomes (Table 1). The densovirus contigs ranged in size from 3,061 to 5,963 nt (5,179.0 ± 684.4 [mean ± standard deviation]). The average sizes of the ORFs found in sea star densovirus were as follows, with standard deviations in parentheses: NS1, 1,694.8 (±33.3) nt; NS2, 883.1 (±28.5) nt; NS3, 807.8 (±109.2) nt; and VP, 2,723.0  (±98.2) nt. The mean pairwise nucleotide identity was greater than amino acid identity among sea star densovirus genomes for NS1, NS3, and VP ORFs (Fig. 3 and Fig. S1). The NS1 ORF had higher sequence conservation (55.7% average nucleotide and 43.2% average amino acid pairwise identity) than the ORFs of NS3 (32.6% average nucleotide and 18.6% amino acid pairwise identity) and VP (43.8% average nucleotide and 34.2% average amino acid pairwise identity). The current delineation for a new parvovirus species is based on the pairwise amino acid sequence identity of NS1. Parvoviruses encoding NS1 proteins with an >85% pairwise amino acid sequence identity are considered the same viral species (18). Using this species definition, 29 new sea star densovirus species were defined from the 39 genotypes discovered. There were 8 viral species that contained 2 or 3 genotypes and 21 species that contained a single genotype (Table 1).

**FIG 4:**
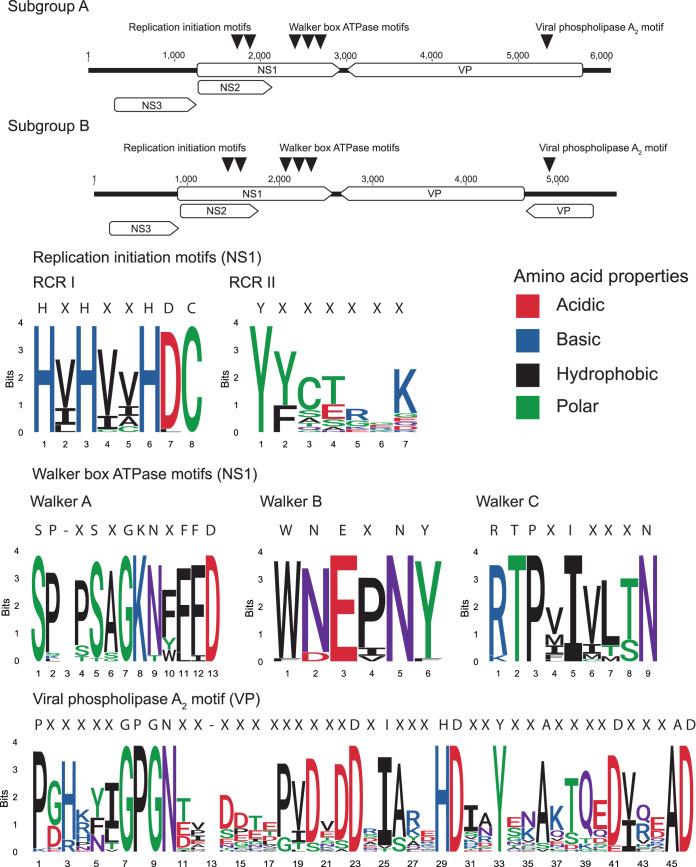
Sea star-associated densoviruses exhibit two genome organizations and contain motifs typical of densoviruses. Triangles indicate positions of amino acid motifs. Consensus sequences above sequence logos are defined by a 90% identity agreement among all sea star-associated densoviruses.

All sea star densoviruses discovered thus far have ambisense genomes that fall into subgroups A and B, which differ only by the VP ORF organization (19) (Fig. 4). The NS1 and VP ORFs identified in this study contain all the expected motifs that are characteristic of densoviruses (20). These motifs include RCR I and RCR II of the replication initiation motifs, Walker A, B, and C of the NTP-binding and helicase motifs, and the viral phospholipase A_2_ motif.

### Identification of EVEs of densovirus origin.

**FIG 5:**
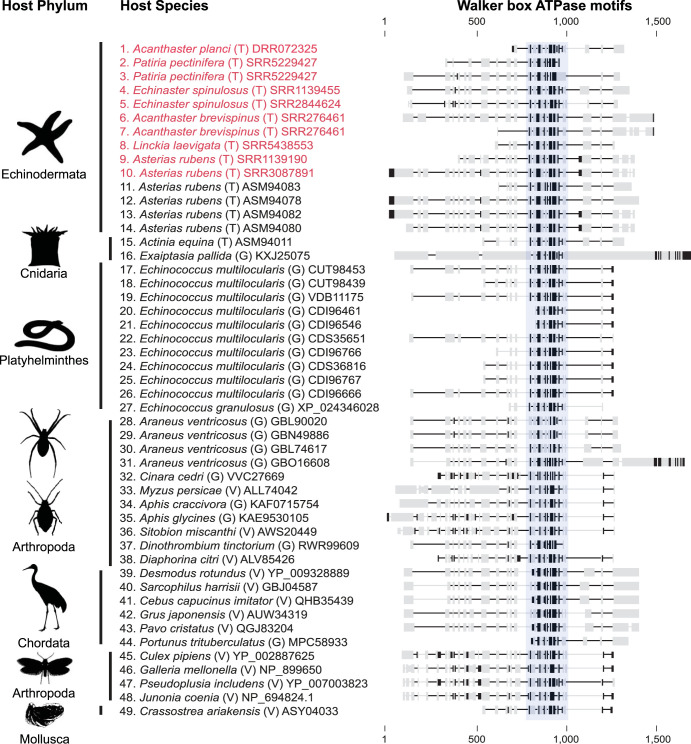
An overview of endogenized densovirus elements (EVEs) illustrating the conserved existence of Walker box ATPase motifs. EVEs found in this study are shown in red. Sequences in alignment are grouped by host phylum. Sequences are labeled by host species and by origin of sequence from host genome (G), extant virus (V), and host transcriptome (T) and NCBI accession number. Amino acids in bold indicate a sequence identity of 75% or greater within the alignment. The blue highlighted region indicates the NTP-binding and helicase region containing Walker A, B, and C motifs found within the NS1/Rep ORF in densoviruses and parvoviruses.

A total of 8 of the 179 transcriptomic libraries contained contigs with densovirus-like sequences. Ten densovirus-like sequences were found among the 8 libraries based on homology searches against the sea star-associated densovirus database. These densovirus-like sequences encoded only part of NS1, lacked RCR motifs, and were not the typical coding length found in sea star-associated densovirus genomes. These sequences are likely endogenized viral elements of densovirus origin due to the fragmentation of the viral genome and the missing enzymatic motifs. The endogenized densovirus elements primarily contained Walker box ATPase motifs with homology to those of parvoviruses and densoviruses from a broad diversity of hosts (Fig. 5 and Table S7). The transcriptomes containing densovirus-like sequences came from the following species: Acanthaster planci (SRA run identifier DRR072325), Patiria pectinifera (SRR5229427), Echinaster spinulosus (SRR1139455 and SRR2844624), Acanthaster brevispinus (SRR9276461), Linckia laevigata (SRR5438553), and Asterias rubens (SRR1139190 and SRR3087891) (Table S6).

## DISCUSSION

The initial investigation for a viral agent associated with SSWS involved viral metagenomic surveys (DNA and RNA) to compare the viral consortia between species and between asymptomatic and symptomatic individuals to determine the most likely viral candidate for further investigation (11). The conclusions from these molecular surveys were that SSaDV was more represented in metagenomic libraries of symptomatic individuals and was present in symptomatic metagenomes prepared from multiple sea star species (11). However, these data show that SSaDV was 1 of 10 densoviruses present and was neither more abundant by number of reads per library comparing asymptomatic to symptomatic individuals (based on read mapping analysis) nor more prevalent between libraries comparing symptomatic to asymptomatic individuals (Fig. 1 and Table S5). This result contradicts the original conclusion from the metagenomic data that SSaDV was associated with SSWS. The difference in results is directly attributable to the difference in the bioinformatic assembly approach. The original analysis took an overlap-layout-consensus global assembly approach using the 28 DNA viral metagenomes not including the 4 RNA viral metagenomes (11). In this study, we chose SPAdes, a more sensitive de Bruijn graph assembler, and included the RNA viral metagenomes in the analysis which contained six of the nine novel densoviruses found in this data set (Table 1). We did find SSaDV to be, on average, the most abundant densovirus by read mapping analysis and, thus, the most consistently assembled, likely biasing its assembly and discovery in the original analysis. The higher representation of SSaDV among metagenomic libraries may be the result of higher viral enrichment in those samples prior to sequencing rather than a result of greater viral loads prior to metagenomic preparation. While a higher abundance of SSaDV could reflect important host-virus biology, it remains to be determined whether greater viral loads, measured by quantitative PCR (qPCR), of SSaDV has any biological significance. A pitfall of viral metagenomics from animal tissue using a viral enrichment method is the significant variability in nonviral genetic material between viromes within a study, making quantitative comparisons difficult (21). According to our metagenomic survey, SSaDV is one species within a diverse extant population of densoviruses present in sea star populations on the West Coast of the United States.

The discovery of densoviruses associated with sea stars collected from China, Antarctica, and the Pacific and Atlantic coasts of the United States indicates their ubiquitous distribution and substantial extant diversity (Table 1, Fig. 3, and Fig. S1). The diversity observed in this study is likely a small fraction of the total diversity among echinoderms, considering that these viruses have also been found in sea urchins (14). Sea star-associated densoviruses also seem to be pervasive in wild populations. The two densovirus genotypes with the best-described ecological characteristics, SSaDV and AfaDV, share striking similarities. Both viral genotypes are not species specific, are found across a large geographic range, are commonly found in asymptomatic individuals, and have a wide tissue tropism, with pyloric caeca being the primary tissue of detection (Fig. 2) (13). This set of characteristics suggests that both viruses form persistent infections in sea stars.

The genus *Ambidensovirus*, to which both previously described sea star-associated densoviruses belonged to based on genome organization, was recently divided into seven newly proposed genera to resolve paraphyly within the genus (8). In this new arrangement, SSaDV and Cherax quadricarinatus (shrimp) densovirus (CqDV; the densovirus most genetically similar to SSaDV prior to the discovery of AfaDV) were assigned to the genus *Aquambidensovirus*, putatively uniting all aquatic densoviruses (8, 13, 22). Our phylogenetic analysis did not support the monophyly of sea star-associated densoviruses within the newly proposed *Aquambidensovirus* genus, nor did all aquatic densoviruses cluster into a single well-supported clade (Fig. 3 and Fig. S1). Newly proposed classification schemes within the *Ambidensovirus* genus would greatly benefit from the inclusion of broader taxonomic sampling before proposing new systematic arrangements of this highly diverse genus.

Given the lack of immortal cell cultures, the discovery of echinoderm densoviruses has been primarily through metagenomics, and that constraint was the motivation for our analysis of transcriptomes as an additional and alternative option for densovirus discovery. Host transcriptomes have been a rich source of viral discovery from eukaryotes and have expanded our knowledge of host associations for many viral groups, including parvoviruses (12, 23, 24). However, we did not find transcriptomes to be an effective method for the purpose of densovirus discovery compared to viral metagenomes, particularly RNA viral metagenomes. This could be due to various methodological reasons. First, the viral metagenomes prepared for this study were enriched for encapsulated nucleic acids with a cDNA enrichment step; in contrast, transcriptomes target mRNA through rRNA depletion and/or through selection for poly(A) tails. Second, to detect DNA viruses from a host transcriptome requires tissue containing an active infection, which may not be detectable without very high sequence depth. The transcribed EVEs found in this study were detected only in transcriptomes larger than 2.4 Gb. Third, the genomes discovered in the RNA viral metagenomes are likely ssDNA that was carried through the RNA extraction process. ssDNA is an uncommon nucleic acid template for nonviral material and a difficult template to remove during RNA extraction. Most commercial kits use DNases that preferentially target double-stranded DNA (dsDNA) and inefficiently cleave ssDNA. Without preferentially targeting mRNA prior to cDNA synthesis in addition to enriching for encapsulated nucleic acid, the chances of picking up ssDNA in a pool of RNA is much higher.

None of the transcriptome-derived densovirus-like sequences appeared to be extant densoviruses based on ORF architecture and motif repertoire. We conclude that these densovirus-like sequences are likely transcribed EVEs present in host cells. EVEs from *Asterias rubens* and *Acanthaster planci* could be traced to their genomes, while our inability to trace others reflects the lack of publicly available host genomes. The putative EVEs present in *Asterias rubens* were nearly identical to those previously reported for the same host (25), though most that we observed had low sequence identity to previously identified EVEs from other invertebrates. It is likely that these EVEs have been established in the germ line of *Asterias rubens*, and our findings corroborate previous work proposing that sea star densoviruses can infect germ line cells (13). The expression of these EVEs in *Asterias rubens* was found to trigger the RNA interference (RNAi) response, specifically the Piwi-dependent pathway (piRNA), signifying that these EVEs are still recognized as foreign and are regulated through the immune system (25). This RNAi response has been widely observed in terrestrial invertebrate genomes containing EVEs descending from densoviruses (26). We expect that the expansion of echinoderm genomes, and corresponding small RNA libraries, will further support this conclusion.

Essentially all observed EVEs retained the Walker box ATPase motifs, which collectively function as a helicase (Fig. 5) (27). This helicase domain belongs to the superfamily III helicases (SF3), which are more broadly grouped as AAA+ ATPases (28). SF3 helicases are encoded only by DNA and RNA viruses, so their presence in cellular genomes must be the result of endogenization (29, 30). The retention of the Walker box ATPase motifs among endogenized densovirus elements has been observed across a diverse range of invertebrate hosts, suggesting a beneficial function for co-opting and possibly maintaining the function of the SF3 helicase (10, 12, 26, 31, 32). The adaptive benefit of a Walker box ATPase-containing EVE has been demonstrated in the pea aphid (Acyrthosiphon pisum), in which wing development was regulated by two modified densovirus NS1 EVEs, which only retained the Walker box ATPase motifs (32). The expression of these two EVEs under crowded conditions initiated wing development, which could be suppressed by knocking down their expression. These results demonstrate that this viral gene can be co-opted by the host to modulate the response of a phenotypically plastic trait to environmental cues. Another plausible hypothesis for EVE function is the ability to enhance or prime the immune system against new infections (26). However, we observed little sequence identity between extant sea star-associated densoviruses and the EVEs in their transcriptomes. These sequence differences suggest that these EVES are unlikely to have a role in priming the piRNA response against new infections.

We employed metagenomic and transcriptomic approaches to explore the diversity of sea star-associated densoviruses while advancing our understanding of the biogeography of SSaDV, the first densovirus found in sea stars. Empirically, we found that viral metagenomes provided a more effective resource for densovirus discovery than host transcriptomes. We discovered 37 new densovirus genomes from sea stars and identified EVEs expressed in host transcriptomes that are of densovirus origin based on detection of the tripartite SF3 helicase domain in these EVEs. Using PCR, we found SSaDV to have a putatively wide tissue tropism, with the pyloric caeca being the most consistent tissue for viral detection. SSaDV was detected across a broad latitudinal range in the northeastern Pacific from southern California to Alaska and found in tissues in nearly all sea star species tested. These results corroborate the hypothesis that these viruses are common among populations and suggest that they form persistent infections in sea stars. Given the diversity of densoviruses and their broad distribution among tissues, populations, and species of both asymptomatic and symptomatic sea stars, we propose that the association of SSaDV with sea star wasting syndrome should be critically reassessed relative to the mounting evidence that this virus may not be the pathogen that causes this disease and instead a common constituent of these animals’ microbiomes.

## MATERIALS AND METHODS

### Tissue collection and DNA extractions.

We collected 887 tissue samples from 660 individual sea stars spanning 12 species from 42 locations from the temperate eastern Pacific coast of the United States from 2005 and 2014 to 2019 (Tables S1, S2, and S3). Thirty samples were collected during the peak of SSWS epidemic observed from mid-2013 to 2015 in the Northeast Pacific (33). The majority of samples collected were from asymptomatic individuals (85% of sea stars sampled). Tissues were collected from sea star specimens by vivisection immediately upon collection followed by flash-freezing in liquid nitrogen and storage at –80°C or –20°C until dissection or were sampled from individuals in the field nonlethally and preserved in RNAlater (Sigma Aldrich) or ethanol (EtOH) (Table S1). Coelomic fluid samples were collected only from vivisected specimens using a 25-gauge 1.5-in. (0.5-mm by 25-mm) needle attached to a 3-ml syringe inserted through the body wall into the coelomic cavity. DNA was extracted from tissues and coelomic fluid using the Zymo Research Quick-DNA Miniprep Plus kit or the Zymo Research Duet DNA/RNA Miniprep kit following the manufacturer’s protocol. DNA was quantified using a NanoDrop or Quant-iT PicoGreen dsDNA assay kit (Invitrogen) (Tables S1 and S3).

### PCR and Sanger sequencing of SSaDV.

Primers were designed targeting the structural gene of SSaDV using Primer3web (version 4.1.0). The primers were as follows: VP1 forward primer (5′-TGGCCACTCATCATGTCTCT-3′) and VP1 reverse primer (5′-CTTGGGGTCCTTCATGAGC-3′). New England BioLabs (NEB) Q5 high-fidelity DNA polymerase was used following the manufacturer’s protocol for a 50-μl reaction volume. Thermal cycling was performed in a Bio-Rad C1000 thermal cycler using the following conditions: initial denaturing at 98°C for 30 s followed by 35 cycles of denaturing (98°C for 10 s), annealing (67°C for 20 s), and extension (72°C for 20 s) and a final extension at 72°C for 2 min. Annealing temperature was based on NEB Tm Calculator recommendation using default primer concentration of 500 nM. The resulting amplicon of the PCR was 534 nucleotides (nt). All PCRs included a positive control, a kit negative control, and PCR reagent negative control to account for false positives and false negatives. A total of 10 to 15 μl of a PCR product was used for gel visualization. The remaining PCR product was processed using a ZR-96 DNA Clean & Concentrator-5 kit (Zymo Research) and submitted to the Cornell Core Biotechnology Resource Center Genomics Facility for DNA sequencing. DNA sequencing was performed on Applied Biosystems automated 3730xl DNA analyzers using BigDye Terminator chemistry and AmpliTaq-FS DNA polymerase.

### Viral metagenome preparation.

Five RNA viral metagenomes were prepared from five different species of sea stars: *Pisaster ochraceus* (*n* = 1), Labidiaster annulatus (*n* = 1), *Leptasterias* sp. (*n* = 1), Mediaster aequalis (*n* = 1), and Neosmilaster georgianus (*n* = 1) (Table S4). The preparation of viral metagenomes followed the method of Hewson et al., modified from that of Thurber et al. (17, 34). Pyloric caeca from sea stars were homogenized in a 10% bleach-cleaned NutriBullet with 0.02-μm-filtered 1× phosphate-buffered saline (PBS). Tissue homogenates were pelleted by centrifugation at 3,000 × *g* for 5 min, and the supernatant was syringe filtered through Millipore Sterivex-GP 0.22-μm polyethersulfone filters into 10% bleach-treated and autoclaved Nalgene Oak Ridge high-speed centrifugation tubes. Filtered homogenates were added to 10% (wt/vol) polyethylene glycol 8000 (PEG 8000) in 0.02-μm-filtered 1× PBS with a final volume of 35 ml and precipitated for 20 h at 4°C. Precipitated nucleic acids, and other cell material, were pelleted by centrifugation at 15,000 × *g* for 30 min. The supernatant was decanted and pellets were resuspended in 2 ml of 0.02-μm-filtered 1× PBS. Half of the sample (1 ml) was treated with 0.2 volume (200 μl) of CHCl_3_, inverted three times, and incubated at room temperature for 10 min. After a brief centrifugation, 800 μl of supernatant was transferred into a 1.5-ml microcentrifuge tube. Samples were treated with 1.5 μl of Turbo DNase (2U/μl; Invitrogen), 1 μl of RNase One (10 U/μl; Thermo Scientific), and 1 μl of Benzonase nuclease (250 U/μl; Millipore Sigma) and incubated at 37°C for 3 h. A total of 0.2 volume (160 μl) of 100 mM EDTA was added to the sample after incubation. Viral RNA was extracted using the ZR viral RNA kit (Zymo Research). RNA was converted into cDNA and amplified using the WTA2 kit (Sigma-Aldrich). Prior to sequencing, samples were processed using a ZR-DNA Clean & Concentrator-5 kit, and DNA was quantified with a Quant-iT PicoGreen dsDNA assay kit. Samples were prepared for Illumina sequencing using the Nextera XT DNA library preparation kit prior to 2 × 250-bp paired-end Illumina MiSeq sequencing at the Cornell Core Biotechnology Resource Center Genomics Facility.

### Metagenome-derived viral genome discovery.

Raw paired-end reads were quality trimmed to remove Illumina adapters and phiX contamination. Reads were merged and normalized to a target depth of 100 and a minimum depth of 1 with an error correction parameter. Read quality filtering, trimming, contamination removal, merging, normalization, and read mapping were performed using the BBtools suite (35). Both merged and unmerged reads were used for *de novo* assembly using the default parameters excluding the read error correction option in SPAdes v3.11.1 (36). Contigs shorter than 3000 nt were discarded after assembly, and the remaining contigs were subjected to tBLASTx against a curated in-house database containing 453 genomes from all nine families of eukaryotic ssDNA viruses. Contigs with significant sequence similarity at an *E* value of <1 × 10^−8^ to a densovirus genome were reviewed in Geneious version 9.1.5 (37). Open reading frames (ORFs) were called in Geneious using a minimum size of 550 nt with a standard genetic code and a start codon of ATG. Hairpin structures were identified using Mfold (38). After verification of the contigs as densovirus sequences, reads were mapped back to contigs with a minimum identity of 0.95 to obtain average read coverage and total reads mapped to contigs (Table S5). All densovirus sequences have been deposited in GenBank (see below). In addition to the 5 viral metagenome libraries sequenced in this study, we reanalyzed 30 DNA and 21 RNA viral metagenomes published elsewhere (NCBI BioProject numbers PRJNA253121, PRJNA417963, and PRJNA637333) using the assembly approach described above (Table S4) (11, 17).

### Sea star transcriptome analysis.

A total of 179 sea star transcriptome-sequenced (RNA-seq) paired-end libraries were downloaded from the NCBI database (Table S6). FastX was used to remove reads with lengths of <50 nt and a quality score of <30 (39). Trimmomatic was used to trim adapters (40). Libraries were assembled using default parameters in Trinity v2.1.1 (41). Assembled contigs were annotated against a protein database of sea star densoviruses using DIAMOND with an E value cutoff of <1 × 10^−5^ (42). Contigs with significant similarity to sequences in the sea star-associated densovirus protein database were isolated, ORFs were called, and amino acid sequences from ORFs were further checked by BLASTp against the NCBI nonredundant database. Top BLAST results for each densovirus-like sequence against the NCBI nonredundant database were downloaded for amino acid MUSCLE alignment and visualized using Geneious (37, 43). To verify if the sequences were from the host, BLASTn was performed querying isolated densovirus-like sequences against available sea star genomes (*Asterias rubens*, NCBI taxid 7604, and *Acanthaster planci*, NCBI taxid 133434).

### Densovirus phylogenetics.

Phylogenetic analysis was performed on 89 densovirus sequences that included 39 sea star-associated densoviruses and 50 densovirus sequences from complete or nearly complete genomes available in the NCBI database. All densovirus sequences included in the phylogeny were from extant viruses (i.e., no endogenized densovirus elements). Amino acid sequences from the NS1 gene were aligned with MUSCLE using default parameters (43). The region of NS1 used for alignment (sequence length of 434.9 ± 40.2 [mean ± standard deviation]) spanned motif I of the replication initiation motifs past Walker C of the Walker box ATPase motifs. Phylogenetic relationships between densovirus genomes were inferred by an LG + G + I + F substitution model selected by smart model selection (SMS) in PhyML 3.0 (44). Branch support was determined by bootstrapping for 100 iterations. The resulting maximum likelihood phylogenetic tree was visualized and annotated using iTOL (45). CD-HIT was used to identify viral species using an 85% amino acid sequence identity of NS1 (18, 46).

### Data availability.

Densovirus genome sequences have been deposited in GenBank under accession numbers MT733013 to MT733051 (Table 1). Raw viral metagenomic sequence data have been deposited under BioProject numbers PRJNA253121, PRJNA417963, and PRJNA637333 (Table S4). Assembled sea star transcriptome data have been deposited in OSF (https://osf.io/bh8cr/?view_only=9287953036274329a89270b8c8a51151) (Table S6).

## Supplementary Material

Supplemental file 1

Supplemental file 2

Supplemental file 3

Supplemental file 4

Supplemental file 5

Supplemental file 6

Supplemental file 7

Supplemental file 8
