# Supplementary material for: Diversity of Sea Star-Associated Densoviruses and Transcribed Endogenous Viral Elements of Densovirus Origin
Source: J Virol. 2020 Dec 9;95(1):e01594-20. doi: 10.1128/JVI.01594-20 (PMC7737747; doi:10.1128/JVI.01594-20)

Supplemental Figure 1: Sea star-associated densoviruses are genetically diverse and are not monophyletic. (A) Maximum likelihood phylogenetic tree of densoviruses based on alignment of amino acid sequences from NS1 gene. Collapsed nodes represent densovirus genera while all other branches belong to the genus *Ambidensovirus*. Red names indicate genomes discovered in this study. White circles represent 90-100% bootstrapped support. (B) Representative densovirus genome showing genome organization. (C) Histograms of nucleotide and amino acid pairwise identity comparisons between all sea star associated densoviruses for NS1, NS3, and VP ORFs. Dotted lines indicate mean pairwise identity.

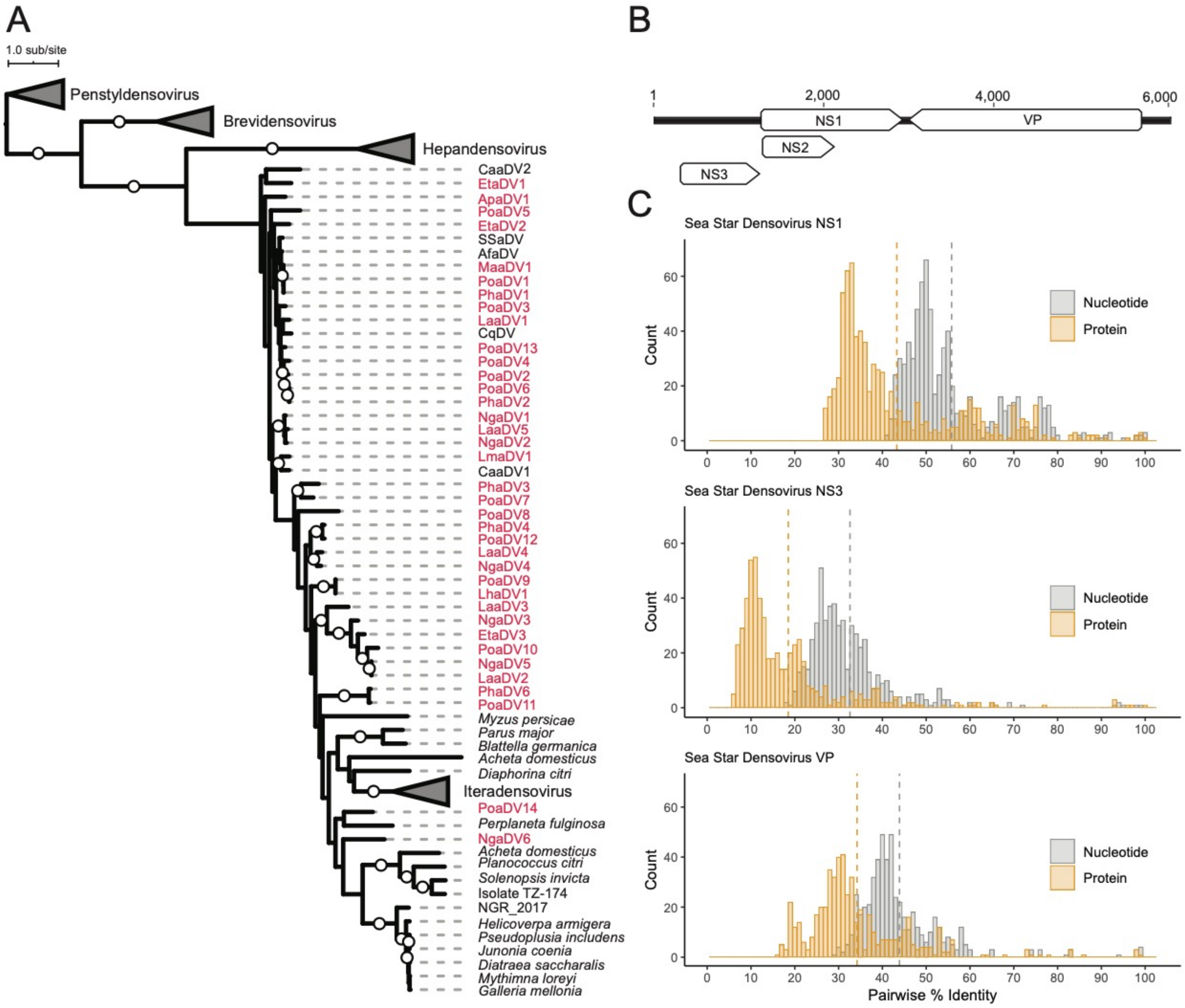

Supplement: Supplemental file 1 [file JVI.01594-20-s0008.pdf]
